# Age and language experience modulate predictive processing in the visual modality

**DOI:** 10.1371/journal.pone.0346695

**Published:** 2026-05-15

**Authors:** Evie A. Malaia, Sean C. Borneman, Joshua Borneman, Julia Krebs, Ronnie B. Wilbur

**Affiliations:** 1 Department of Speech, Language, and Hearing Sciences, University of Alabama, Tuscaloosa, Alabama, United States of America; 2 Department of Physics, Carnegie Mellon University, Pittsburgh, Pennsylvania, United States of America; 3 Department of Linguistics, Purdue University, West Lafayette, Indiana, United States of America; 4 Department of Linguistics, University of Salzburg, Salzburg, Austria; Stellenbosch University, SOUTH AFRICA

## Abstract

Human language processing relies on the brain’s ability to predict structured input rapidly in time. While predictive coding has been widely studied in auditory language users, less is known about how visual language experience influences these mechanisms across their lifetime. We used electroencephalography (EEG) to examine how age and lifelong use of a signed language modulate neural prediction in the visual modality. We recorded EEG from Deaf signers of Austrian Sign Language (ÖGS) while they viewed videos of linguistic and non-linguistic biological motion. Signal complexity and neural coherence to optical flow in the stimuli were computed, and multivariate feature selection was applied to identify age-related patterns. Our results show that neural responses to linguistic motion are characterized by age-dependent increases in coherence to stimuli and changes in signal entropy, suggesting that visual linguistic experience refines predictive mechanisms over time. These effects were strongest in fronto-central and parietal regions, consistent with hierarchical sensory integration for language processing. This study demonstrates that predictive coding in the brain is shaped by modality-specific language experience and continues to evolve with age. The findings offer a new perspective on how sensory and linguistic systems interact to support inference in real-time visual processing.

## 1. Introduction‌‌

Across the lifespan, the human brain undergoes functional reorganization that reflects both developmental adaptation and experience-dependent change. One of the central goals of neuroscience is to understand how the brain anticipates and adapts to structured input. Predictive processing frameworks suggest that the brain continuously generates expectations about incoming sensory data. One influential theoretical account, the Free Energy Principle (FEP) [1], proposes that the brain is an inference engine: it actively predicts sensory input based on prior experience, reducing surprise through hierarchical generative models. In this view, perception is shaped not only by incoming stimuli, but also by top-down expectations grounded in past exposure. Language comprehension, as a high-level cognitive function, provides a natural domain in which to examine such predictive mechanisms.

### 1.1. Neural activity characterizing predictive processing

Predictive processing frameworks propose that perception emerges not merely from bottom-up sensory analysis, but through the continuous interplay between ascending sensory signals and descending predictions generated by internal models [2]. The Free Energy Principle (FEP) models the brain as a hierarchical inference engine that actively predicts incoming sensory data based on learned statistical regularities, continuously updating these predictions to minimize prediction error [1]. In this framework, perception (including perception of language) is fundamentally anticipatory: the brain generates expectations at multiple timescales, from milliseconds to seconds, and compares these predictions against actual sensory input. When predictions match the input (low prediction error), processing is efficient and requires minimal computational resources. When predictions fail (high prediction error), the system updates its models through error-driven learning. An important feature of the model is that the balance between relying on top-down predictions versus bottom-up sensory evidence is thought to shift with experience: as internal models become more refined through exposure to structured input, the brain can rely more heavily on prediction and less on detailed sensory analysis. Electroencephalography (EEG) can provide a window into predictive neural dynamics through the measurement of neural oscillations: fluctuations in electrical activity that reflect synchronized firing patterns across neural populations. The temporal structure of these oscillations is thought to organize information processing across multiple hierarchical levels [3,4]. Low-frequency oscillations (delta: 1–4 Hz, theta: 4–8 Hz) in particular have been implicated in both tracking and predicting slower temporal structures in sensory input, such as phrasal and sentence-level information in language [3]. The relationship between neural oscillations and stimulus structure can be quantified through coherence analysis, which measures the consistency of phase relationships between brain activity and external stimuli. Coherence, in this case, is a direct measure of neural entrainment: the degree to which brain rhythms synchronize with temporal regularities in the stimuli. From a predictive coding (FEP) perspective, enhanced coherence at specific frequencies suggests that the brain has learned to anticipate temporal structure at those timescales, generating oscillatory predictions that align with expected input patterns [1]. Research on spoken language comprehension has extensively documented such predictive entrainment. Neural oscillations in the delta and theta frequency ranges track the amplitude envelope of speech signals, with phase-locking to syllabic and prosodic rhythms facilitating word recognition and syntactic parsing [4]. This entrainment, however, is not merely stimulus-driven: it is modulated by linguistic knowledge, context, and predictability of the stimuli [5]. For instance, neural tracking of speech increases for intelligible versus unintelligible speech [6,7], and for predictable versus unpredictable linguistic sequences [8], suggesting that coherence reflects active prediction rather than passive following. Moreover, aging and experience affect these patterns: older adults show altered neural tracking of speech [9,10], and language proficiency modulates entrainment strength in second-language users [11,12]. These findings establish neural-stimulus coherence as a marker of experience-dependent predictive processing. Much less is known about how predictive processing operates in the visual modality, particularly for complex, structured visual input such as sign language (SL). While visual motion processing has been extensively studied using simple stimuli (e.g., drifting gratings, dot motion), naturalistic visual language presents unique challenges and opportunities for understanding prediction. Unlike auditory speech, where acoustic measures provide a simple temporal structure for entrainment analysis, sign languages encode linguistic information through multidimensional kinematic patterns, which include hand and face shapes and position, velocity and acceleration patterns, 3D spatial trajectories, and temporal coordination across multiple articulators (two hands, face, body). In sign languages, these motion dynamics carry phonological distinctions (e.g., straight vs. circular paths), morphological information (e.g., aspectual marking through movement repetition or speed modulation), and prosodic structure (e.g., phrase boundaries marked by holds and decelerations) [13,14]. The temporal organization of these features operates across multiple timescales simultaneously: from rapid articulator movements (8–15 Hz) to syllable-like sign units (4–5 Hz) to phrasal and sentence-level structures (0.5-2 Hz) [15–17]. This hierarchical temporal organization makes sign languages a rich testbed for examining multi-scale predictive processing in the visual domain.

### 1.2. Visual language and predictive processing

Sign languages present a unique opportunity to examine how lifelong linguistic experience shapes predictive mechanisms in the visual modality, independent of auditory processing. Deaf individuals who acquire sign language as their first language develop highly specialized neural systems for processing structured visual motion. Neuroimaging studies have shown that sign language comprehension recruits a left-lateralized fronto-temporal network similar to that engaged during spoken language processing [18–20], despite the difference in sensory modality. This suggests that linguistic computation drives core language network organization. Research using biological motion has demonstrated that Deaf signers process hand and body movements differently from non-signers, showing enhanced sensitivity to linguistically-relevant kinematic parameters and distinct patterns of neural activation in posterior temporal and parietal cortices [21,22]. These regions are thought to support motion prediction and temporal sequence processing, suggesting experience-dependent reorganization of visual prediction systems. Recent work has begun to examine neural entrainment to sign language using EEG coherence analysis. Our prior research demonstrated that Deaf signers exhibit frequency-specific neural tracking of optical flow (a measure of visual motion dynamics) in sign language videos [23,24]. Specifically, coherence between EEG signals and the temporal profile of visual motion was strongest in low-frequency bands, and localized to frontal and fronto-central scalp regions associated with language processing. This coherence pattern was specific to linguistically structured motion; when signers are presented with non-linguistic stimuli, the coherence pattern is substantially reduced or absent. This suggests that neural entrainment reflects not just frequency-following response in the visual domain, but predictive engagement with the temporal structure of linguistic units. No studies have yet examined how such predictive dynamics evolve across the lifespan in experienced sign language users, or how long-term experience shapes predictive neural mechanisms in the visual domain.

### 1.3. Aging effects on predictive processing

Aging introduces systematic changes to neural structure and function that have important implications for predictive processing. One well-documented phenomenon is the alteration of EEG spectral properties with advancing age, particularly an increase in spectral flatness—the flattening of the power spectrum such that power becomes more evenly distributed across frequencies [25] This flattening reflects what has been termed “neural noise”: increased randomness in neural firing patterns, reduced synchronization between neural populations, and weakened phase-amplitude coupling [26,27]. From an information-processing perspective, increased neural noise degrades neural representations and reduces the efficiency of neural communication. In the context of predictive coding, this creates a challenge: noisy neural signals make it difficult to maintain precise temporal predictions. Older adults, then, may struggle to generate or maintain accurate predictions about sensory input, particularly when the input has complex temporal structure. However, aging does not affect all cognitive systems uniformly. While structural measures such as spectral flatness show consistent age-related declines, functional measures of task performance often remain stable or even improve in domains where individuals have extensive experience [28]. This suggests that lifelong learning can compensate for age-related increase in neural noise, at least partially [10]. In the domain of language, older adults show preserved or enhanced use of contextual information and world knowledge to facilitate comprehension, even as lower-level perceptual abilities decline [29,30]. From a predictive processing perspective, this pattern is consistent with increased reliance on top-down predictions (built through decades of experience) to compensate for degraded bottom-up sensory signals. For Deaf signers with extensive, long-term use of sign language as their primary mode of communication, we might therefore expect a complex interaction between age-related neural changes (e.g., increased spectral flatness) and experience-dependent enhancement of visual-linguistic prediction. Specifically, while general spectral properties may show age-related decline, the specific neural mechanisms that track and predict sign language structure may be preserved or even enhanced, reflecting cumulative linguistic experience.

The present study investigates how lifelong visual language experience and age-related neural changes affect predictive processing in the visual-linguistic domain. We recorded EEG from Deaf users of Austrian Sign Language (ÖGS) spanning a wide age range (28–68 years) while they viewed videos of natural sign language sentences and time-reversed control stimuli. Time-reversed videos serve as a critical control: they contain identical visual motion statistics (same amount of movement, same velocity distributions) but lack interpretable linguistic structure. By comparing neural responses to forward versus reversed signing, we can isolate effects that are specific to linguistic prediction rather than general visual motion processing. We analyzed both the spectral properties of EEG, which provide information about general neural signal organization, and the coherence between EEG and visual motion (optical flow) in the stimuli, which provides measures of neural entrainment to temporal structure [23,31]. We pose three specific research questions:

1) How does the strength and timing of neural entrainment to visual motion vary depending on whether the stimulus is linguistically structured (sign language) or not (time-reversed videos)?
2) How are these coherence patterns localized in the brain—particularly between frontal and posterior regions known to support top-down and bottom-up processing, respectively?
3) How do these coherence features change with age, reflecting both structural decline and the cumulative influence of lifelong language experience?

If visual language experience refines predictive mechanisms, we would expect age to be associated with increased coherence specifically during sign language processing (reflecting better-tuned predictions), potentially in posterior regions associated with visual motion analysis. Alternatively, we might observe age-related increases in response lag (time-shift) during processing of non-linguistic stimuli, reflecting difficulty in generating predictions for unpredictable input. By examining both general spectral features (which capture structural neural changes) and stimulus-specific coherence patterns (which capture functional predictive engagement), we can dissociate age-related decline from experience-dependent changes in visual linguistic prediction.

## 2. Materials and methods

### 2.1. Participants

Individuals proficient in Austrian Sign Language (ÖGS) were recruited for the study. All participants reported normal or corrected-to-normal vision and had no history of neurological disorders. Their language proficiency was assessed through conversational interviews conducted by a certified Austrian Sign Language interpreter with 10 years of professional experience. This represents the gold standard for proficiency assessment in this linguistic community, as standardized psychometric tests for ÖGS proficiency do not currently exist. Participants varied in their age of acquisition of ÖGS; the majority were deaf from birth or early childhood, while a small number acquired ÖGS later in life. All participants were verified as proficient, active members of Austrian Deaf community through conversational interview with a certified interpreter. Because our analyses treat age as a proxy for cumulative linguistic experience, the relevant criterion is long-term, active use of ÖGS as a primary language; this criterion is met by all participants.

The study included 24 participants (13 male), aged between 28 and 68 years (M = 42, SD = 12.27). All procedures were approved by the University of Salzburg Ethics Commission (Protocol EK-GZ: 07/2015). Recruitment began on November 1, 2015, and ended May 31, 2016; Written informed consent was obtained from all participants prior to participation.

### 2.2. Stimuli and procedure

Each participant was presented with a set of video stimuli comprising 40 sentences signed fluently in ÖGS and their corresponding time-reversed versions, which were not linguistically interpretable. Additionally, 200 filler videos were included, consisting of sentences with classifier constructions, topicalized sentences in Subject-Object-Verb (SOV) and Object-Subject-Verb (OSV) word orders, and simple SOV-structured sentences. Neural and behavioral responses to filler videos, aimed at preventing habituation, are discussed in detail in [32,33]. Behavioral responses to fillers paralleled those for time-forward videos but were not included in the primary analysis due to differing spectro-temporal properties.

**Fig 1:**
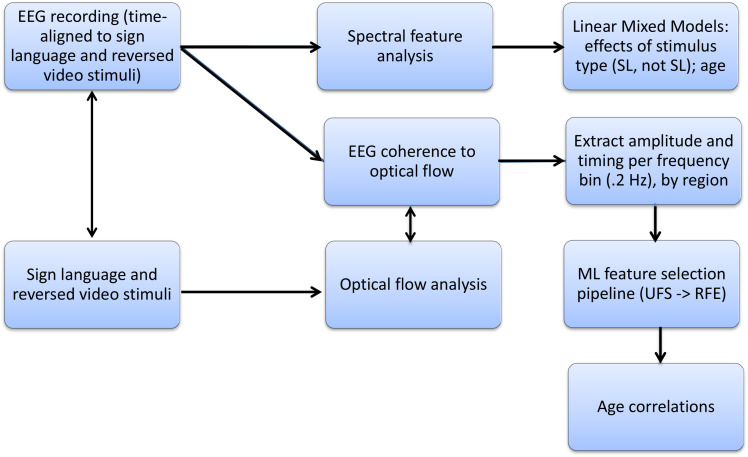
Analysis pipeline. EEG recordings time-aligned to sign language and time-reversed video stimuli were processed along two parallel streams: (top) spectral feature analysis submitted to linear mixed-effects models examining effects of stimulus type and age; and (bottom) coherence between EEG and optical flow of the stimuli, with amplitude and timing features selected using ML pipeline (UFS → RFE) prior to age correlation analyses.

The sign language stimuli were presented as dynamic videos of signed sentences comprehensible to fluent signers (see Fig 1). A list of glossed sentence translations is provided in the appendix. To generate non-linguistic stimuli while maintaining identical spatiotemporal properties, videos were time-reversed. Participants rated the acceptability of each stimulus, including reversed videos, on a 7-point scale (1 = “not ÖGS,” 7 = “good ÖGS,” with 4 indicating “not ÖGS, but understandable”).

Stimulus conditions were pseudo-randomized, ensuring that no condition appeared more than twice consecutively. Two balanced pseudo-random sequences were used across participants. Prior to the experiment, participants completed a training session to familiarize themselves with the task. Videos were displayed on a 35.3 x 20 cm screen at a resolution of 1280 x 720 pixels. Participants were instructed to minimize movement during video presentations. Each trial began with a fixation cross (2000 ms), followed by a 200 ms blank screen, and then the stimulus video. After the video, a question mark appeared for 3000 ms, prompting participants to rate the stimulus using a keyboard response.

### 2.3. EEG data acquisition and processing

EEG data were collected using a 26-channel system at a sampling rate of 500 Hz with active electrodes positioned according to the 10/20 system (Fz, Cz, Pz, Oz, F3/4, F7/8, FC1/2, FC5/6, T7/8, C3/4, CP1/2, CP5/6, P3/7, P4/8, O1/2). Electrodes were secured with an elastic cap (Easy Cap, Herrsching-Breitbrunn, Germany), and impedance was maintained below 5 kΩ. Eye movements were recorded via electrodes placed at the outer canthi (HEOG) and superior/inferior orbital ridges (VEOG). AFz served as the ground electrode, and all recordings were referenced to the left mastoid.

During each trial, numerical triggers synchronized stimulus onset with EEG recordings. Offline processing included re-referencing to the average of both mastoids, and ocular artifact correction using the Gratton and Coles method [34]. EEG data were segmented from stimulus onset to 5 seconds post-onset, ensuring the analysis focused on responses to ongoing video stimuli (see Fig 1). While scalp EEG reflects volume-conducted activity from distributed neural sources, the regional organization of our electrode groups permitted inference about broad functional-anatomical patterns.

## 3. Analysis

To examine how aging and sign language experience shape neural dynamics during visual language processing, we conducted two complementary types of analyses.

First, we performed spectral analysis of EEG signals recorded in response to sign language and reversed video stimuli. This analysis quantifies the statistical properties of neural activity — such as spectral entropy and flatness — across brain regions. These measures are used in cognitive neuroscience to assess signal complexity and functional organization (cf. [25]), and can be directly compared to existing literature on aging-related changes in brain electrophysiology. This analysis was conducted on raw EEG recordings collected during exposure to both natural signed stimuli and their time-reversed counterparts.

Second, we investigated the brain’s entrainment to dynamic visual input. To quantify the spatiotemporal dynamics of motion in the stimulus videos, we used optical flow analysis, which estimates local motion vectors between consecutive frames based on pixel-intensity changes [35,36]. This method provides a continuous and objective representation of visual motion analogous in temporal resolution to raw video, while specifically isolating kinematic information such as velocity. Compared to manually coded linguistic annotations, which mark only discrete events (e.g., sign onsets or holds), optical flow captures the full continuous dynamics of articulatory movement, including low-frequency fluctuations (such as body position changes) that are critical for sign language signal [13]. Unlike raw pixel-based representations, optical flow abstracts away irrelevant visual properties such as luminance or color, making it computationally optimal for coherence analysis with high-density EEG data. Prior work in sign language research has demonstrated that optical flow–derived metrics reliably predict neural responses in signers and accurately capture the information transfer capacity of sign articulators [31,37], providing optimal balance between ecological validity, kinematic specificity, and analytical feasibility for investigating neural entrainment to visually structured linguistic input. Coherence was computed in terms of both amplitude (i.e., strength of coupling) and timeshift (i.e., temporal lag between stimulus motion and neural response) across multiple frequency bins and scalp regions. This approach yielded a high-dimensional set of features (n = 496), reflecting frequency-specific stimulus tracking across conditions.

Given the large number of coherence features, and our specific interest in how Sign Language processing and non-linguistic dynamic perception interact with aging, we applied a machine learning-based feature selection pipeline. First, we classified signed versus non-signed (reversed) stimuli using coherence and timeshift features, identifying those that best distinguish between linguistic and non-linguistic visual input. Then, we focused age-related correlation analyses only on this subset of informative features. This two-step approach allowed us, first, to isolate aspects of neural entrainment that are relevant to sign language comprehension, and second, to examine how their relationship to age varies across brain regions and frequencies.

### 3.1. Spectral analysis of EEG signals

Spectral analysis was conducted to characterize the statistical properties of the EEG signal in the frequency domain, specifically focusing on measures of spectral complexity and energy distribution, separately for each of the regions of interest: frontal, posterior, left, and right hemispheres. The anterior region included channels FC1, FC2, F3, F4, and Fz. The posterior region comprised P3, P4, P7, P8, and Pz. The left hemisphere was defined by FC5, C3, CP1, CP5, and T7, while the right hemisphere included FC6, C4, CP2, CP6, and T8. The analysis included the computation of spectral entropy, spectral centroid, spectral spread, and spectral flatness. EEG data, which included 5-second segments time-locked to stimuli onsets at 500 Hz recording frequency, yielded frequency resolution .2 to 250 Hz.

Spectral descriptors were computed in MATLAB (The MathWorks, Inc.) utilizing the spectralEntropy, spectralCentroid, spectralSpread, and spectralFlatness functions, applied to the power spectral density (PSD) of each EEG segment. The PSD was estimated using a rectangular window.

Spectral entropy (*H*) was computed as a measure of the signal’s spectral complexity, defined by:


$$\begin{array}{c} H=-\frac{\sum_{i=1}^{N}P(f_{i})\log_{2}P(f_{i})}{\log_{2}N}, \end{array}$$ (1)

where *P*(*f*_*i*_) is the normalized power at frequency bin *f*_*i*_, and *N* is the total number of frequency bins. Higher entropy values indicate a flatter spectral distribution, reflecting increased signal unpredictability.

The spectral centroid (*C*) was calculated as the weighted mean of the frequency components, providing an estimate of the spectral “center of mass,” according to:


$$\begin{array}{c} C=\frac{\sum_{i=1}^{N}f_{i}\cdot P(f_{i})}{\sum_{i=1}^{N}P(f_{i})}. \end{array}$$ (2)

Spectral spread (*S*) quantified the dispersion of the power spectrum around the spectral centroid, computed as:


$$\begin{array}{c} S=\sqrt{\frac{\sum_{i=1}^{N}(f_{i}-C{)}^{2}\cdot P(f_{i})}{\sum_{i=1}^{N}P(f_{i})}}. \end{array}$$ (3)

Spectral flatness (*F*) was defined canonically, as the ratio of the geometric mean to the arithmetic mean of the power spectrum:


$$\begin{array}{c} F=\frac{{\left(\prod_{i=1}^{N}P(f_{i})\right)}^{\frac{1}{N}}}{\frac{1}{N}\sum_{i=1}^{N}P(f_{i})}. \end{array}$$ (4)

All spectral features were computed for each EEG epoch and electrode channel, and subsequently averaged across trials to yield participant-level indices of spectral properties. These measures were then used in statistical analyses to investigate age- and experience-related changes in neural signal complexity and organization.

### 3.2. Optical flow analysis and coherence computation

Optical flow, a computer vision technique, was used to quantify motion between consecutive video frames. This measure tracks signal variation over time by calculating velocity magnitude (in pixels per frame) based on edge contrast. Optical flow preserves both spatial and temporal information, producing a velocity profile for each frame.

Optical flow computations were performed using MATLAB’s vision toolbox, yielding velocity matrices equivalent in size to the video frames. Histograms of velocity distributions were generated for each frame, and total motion magnitude was computed by summing velocity amplitudes across bins, resulting in an optical flow time series.

Coherence between the optical flow time series and EEG data was assessed at frequencies from .02 Hz to 12.5 Hz (limited by the 25 fps video frame rate). Timeseries were filtered at each frequency using a second-order IIR bandpass filter. Canonical correlation analysis, implemented via MATLAB’s NoiseTools toolbox [38], was employed to compute coherence between EEG signals and optical flow. Peak correlation values and corresponding time shifts were extracted for each frequency, stimulus, and electrode site. Sensor data was averaged over regions of the scalp (Anterior, Posterior, Left, Right) for each condition and participant. Anterior region included electrodes F3, F4, Fz, FC1, and FC2; posterior region included electrodes P7, P8, P3, P4, Pz; left hemisphere region included electrodes C3, FC5, T7, CP1, CP5; right hemisphere region included electrode channels C4, FC6, T8, CP2, CP6. This procedure produced the matrix with 496 features (4 regions x 62 frequency bins for peak correlation, and 4 regions x 62 bins for timeshift).

### 3.3. Feature selection pipeline

The coherence analysis generated 496 features (4 regions × 62 frequencies × 2 measures). Rather than testing each feature independently, which would require extensive multiple-comparison correction and severely reduce statistical power—we used a machine learning–based feature selection approach to identify the subset of features most informative for distinguishing linguistic from non-linguistic processing. This procedure served two complementary goals: (1) dimensionality reduction by isolating features that contributed meaningfully to stimulus discrimination, and (2) neurobiological interpretability by focusing subsequent analyses on neurally relevant signal components rather than noise. Conceptually, this is equivalent to defining functionally derived regions of interest in fMRI studies prior to hypothesis testing.

To identify neural coherence features predictive of age, we implemented a three-step feature selection and model interpretation pipeline. This approach was applied to coherence metrics computed between EEG signals and optical flow of sign language stimuli, including both time-shift and amplitude features across brain regions, using Python and the scikit-learn library. A preliminary application of this pipeline focusing on coherence-based classification of linguistic versus non-linguistic stimuli is reported in [39].

#### 3.3.1. Univariate feature screening (UFS).

As an initial filtering step, we performed univariate feature screening (UFS) using mutual information regression with age as the target variable. This analysis was conducted separately for three subsets of features: (1) time-shift features, reflecting the delay in neural response relative to visual motion; (2) amplitude features, representing signal coherence strength; and (3) spatial features corresponding to electrode locations. Features exhibiting minimal mutual information with age were excluded from subsequent analyses.

#### 3.3.2. Recursive feature elimination (RFE).

Following UFS, we applied recursive feature elimination (RFE) to further refine the feature set. A tree-based regressor (Random Forest) was trained to predict participant age using the remaining features. Feature importance was estimated from the fitted model, and the weakest features were recursively removed. This method accounts for potential non-linear relationships and interactions among features. Additionally, hierarchical feature grouping was considered based on anatomical region and frequency band, with group-level elimination performed where appropriate.

#### 3.3.3. Statistical correlation analyses.

To assess age-related differences in neural coherence features, we computed Pearson correlation coefficients between participant age and each retained feature following UFS and RFE steps. Correlations were assessed separately for the Sign Language (Condition 1) and reversed video (Condition 0) datasets. Statistical significance was evaluated at α=.05, without correction for multiple comparisons, given the exploratory nature of the analysis.

#### 3.3.4. Spectral analysis and modelling.

Spectral features (entropy, flatness, centroid, spread) were analyzed separately across four anatomically defined electrode regions. Each feature quantifies a distinct property of the EEG signal’s frequency structure rather than time-varying power, as would be captured in a spectrogram. For instance, spectral centroid reflects the frequency “center of mass” of the power spectrum (analogous to acoustic brightness), spectral spread indexes bandwidth around that centroid, spectral flatness distinguishes narrowband or tonal signals from broadband or noise-like ones, and spectral entropy measures the overall disorder or uniformity of the spectrum. These descriptors are complementary and non-redundant: they summarize different aspects of signal organization within the analysis window rather than testing the same hypothesis multiple times.

Separate models were fitted for each region and feature because spectral features capture neural activity differences emerging at distinct timescales (task-driven versus experience-dependent) and because region-specific effects are theoretically and anatomically meaningful. We fitted 16 separate linear mixed-effects models: one model for each spectral feature (4 features: Spectral Entropy, Spectral Centroid, Spectral Spread, Spectral Flatness), separately for each brain region (4 regions: Frontal, Posterior, Left hemisphere, Right hemisphere). Models were fitted using maximum likelihood (ML) estimation. Subject-specific random intercepts accounted for baseline differences in spectral properties across individuals. No random slopes were modeled. The model for each feature *f* in region *r* was specified as:


$$\begin{array}{c} f_{ij}=\beta_{0}+\beta_{1}({\text{Age}}_{i})+\beta_{2}({\text{Stimulus}}_{j})+\beta_{3}({\text{Age}}_{i}\times {\text{Stimulus}}_{j})+u_{i}+\varepsilon_{ij} \end{array}$$ (5)

where *f*_*ij*_ is the spectral feature value for participant *i* on trial *j*, $\beta_{0}$ is the fixed intercept, $\beta_{1}$ through $\beta_{3}$ are fixed effects for Age, Stimulus, and their interaction, *u*_*i*_ is the random intercept for participant *i*, and $\varepsilon_{ij}$ is the residual error. Stimulus was treatment-coded with Reversed video as the reference level (coded 0), such that $\beta_{2}$ reflects the difference in each spectral feature during Sign Language viewing relative to Reversed video viewing.

A summary of significant effects observed in the 16 separate linear mixed-effects models is provided in Table 1; all results for each model, including non-significant effects, are provided in S1 Table. To confirm that the region-specific patterns reported were not artifacts of analyzing ROIs separately, four additional omnibus models were fitted — one per spectral feature — including ROI as a fixed factor alongside Age, Stimulus, and all two- and three-way interactions; results are reported in S2 Table.

**Table 1:** Summary of significant spectral effects of Age and Stimulus across brain regions.

Predictor	Region	Feature	β	SE	$\chi^{2}$	*p*	AIC	BIC
Age	Frontal	Spectral Flatness	.00003	.00001	11.299	<.001	−14110.1	−14078.2
	Posterior	Spectral Flatness	.00003	.00001	10.888	<.001	−14220.9	−14188.9
	Left	Spectral Flatness	.00003	.00001	11.057	<.001	−14208.8	−14176.8
	Right	Spectral Flatness	.00003	.00001	8.639	.003	−14114.9	−14082.9
Stimulus	Frontal	Spectral Entropy	−.016	.008	17.894	<.001	−3556.2	−3524.3
	Posterior	Spectral Entropy	−.014	.007	22.743	<.001	−3550.8	−3518.8
	Left	Spectral Entropy	−.019	.008	38.682	<.001	−3525.1	−3493.1
	Left	Spectral Spread	−.436	.203	4.746	.029	+6420.4	+6452.3
	Left	Spectral Centroid	−.683	.308	25.464	<.001	+7680.8	+7712.7
	Right	Spectral Entropy	−.016	.008	21.191	<.001	−3563.0	−3531.1
	Right	Spectral Centroid	−.466	.276	16.946	<.001	+7356.4	+7388.4

*Note.* Only significant effects are shown. β = fixed effect estimate; SE = standard error; $\chi^{2}$ = Type II likelihood ratio test statistic; AIC = Akaike Information Criterion; BIC = Bayesian Information Criterion (rounded to 1 decimal place). Complete results for all 16 models (4 spectral features × 4 regions), including non-significant effects and random effects variance components, are provided in S1 Table.

## 4. Results

We begin by presenting behavioral results, which serve to anchor the neural data in participants’ perceptual experiences. While stimulus conditions were clearly defined (sign language vs. time-reversed), behavioral ratings provide an independent measure confirming that participants reliably distinguished between linguistic and non-linguistic input.

We then report results from spectral analysis of the raw EEG signal, which evaluates the effects of age, stimulus type, and their potential interaction on the complexity and organization of neural activity across brain regions.

Finally, we turn to coherence between EEG and the optical flow of the stimuli, capturing frequency-specific neural entrainment to visual motion. Given the high dimensionality of these coherence features, we first used machine learning classification to identify those most informative for distinguishing sign language from reversed stimuli. Since stimulus type is already incorporated into the classification stage, we then restricted our statistical analysis to age-related effects among the selected coherence features—focusing on those aspects of neural entrainment that are most relevant for SL comprehension.

### 4.1. Behavioral results

**Fig 2:**
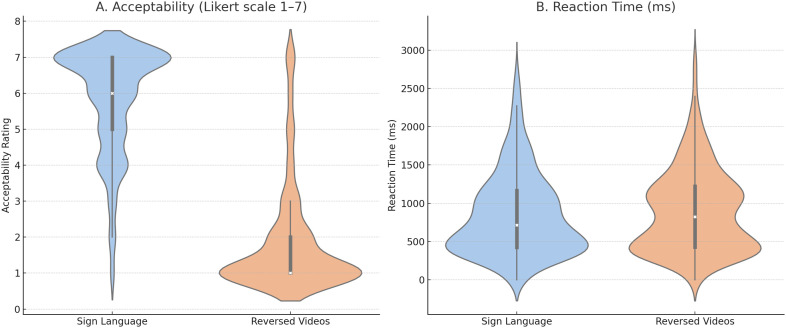
Behavioral results for sign language and time-reversed video conditions. **(A)** Acceptability ratings (7-point Likert scale) were significantly higher for sign language than reversed videos. **(B)** Reaction times did not differ significantly between conditions.

Behavioral ratings collected using a 7-point Likert scale (7 = ‘that is good ÖGS’; 4 = ‘not ÖGS, but understandable’; 1 = ‘that is not ÖGS’) demonstrated a pronounced distinction in participants’ perceptions of the stimuli. Only the time-direct sign language videos were judged to be linguistically acceptable, while the reversed videos were overwhelmingly rated as lacking any resemblance to meaningful sign language. This categorical difference is reflected in the mean ratings, with time-direct videos receiving high scores (*M* = 5.80, *SD* = 1.48), while reversed videos were rated near the bottom of the scale (*M* = 1.72, *SD* = 1.35; see Fig 2. A paired-samples *t*-test confirmed a significant difference in ratings between conditions (*t*(23) = 14.01, *p* < .001), underscoring the drastic perceptual separation between the two types of stimuli. Importantly, response times did not significantly differ between conditions (*t*(23) = −1.3, *p* > .2), wi*t*h comparable latencies observed for time-direct (*M* = 883 ms, *SD* = 535 ms) and reversed videos (*M* = 925 ms, *SD* = 541 ms).

### 4.2. Spectral analysis results

#### 4.2.1. Frontal region.

Spectral flatness revealed a significant fixed effect of Age, β = .0000277, *p* < .001, with older participants showing increased flatness values, suggesting greater spectral uniformity with age.

Regarding Stimulus effects, spectral entropy exhibited a significant fixed effect, β = –.016, *p* < .001, indicating that entropy was higher during Sign Language stimuli compared to reversed stimuli. Stimulus did not reach significance for spectral flatness (β = .000144, *p* = .099).

#### 4.2.2. Posterior region.

Spectral flatness exhibited a significant fixed effect of Age, β = .0000316, *p* < .001, with flatness increasing with age. This suggests age-related increases in spectral uniformity in the posterior cortex. No significant age effects were observed for entropy (*p* = .341).

For Stimulus, posterior spectral entropy demonstrated a significant fixed effect, β = –.014, *p* < .001, with higher entropy during Sign Language than reversed input. Stimulus did not significantly affect spectral flatness.

#### 4.2.3. Left hemisphere.

Spectral flatness showed a significant fixed effect of Age, β = 3.222 × 10^−5^, *p* < .001, indicating increasing flatness in older adults. No age effects were observed for entropy (*p* = 0.784), spread, or centroid.

In terms of Stimulus, spectral entropy revealed a significant fixed effect, β = –.019, *p* = .016, with entropy higher during Sign Language viewing. Spectral spread and spectral centroid also demonstrated significant stimulus effects (β = –.436, *p* = .032; β = –.683, *p* = .027, respectively), indicating altered frequency distribution during linguistic input.

#### 4.2.4. Right hemisphere.

Spectral flatness revealed a significant fixed effect of Age, β = 2.830 × 10^−5^, *p* = .003, mirroring patterns observed in other scalp regions. No significant age effects were found for entropy (*p* = .535) or other spectral measures.

For Stimulus, spectral entropy demonstrated a significant fixed effect, β = –.016, *p* = .037, with higher entropy during Sign Language compared to reversed input. Spectral centroid showed a significant omnibus test ($\chi^{2}$ = 16.946, *p* < .001), although the corresponding regression coefficient narrowly missed significance (β = –.466, *p* = .092).

### 4.3. Interim summary: Spectral data

**Age Effects.** Across scalp regions, age was consistently associated with increased spectral flatness, suggesting greater spectral uniformity in older participants. This trend—interpreted as a form of neural dedifferentiation—was observed in the frontal region (β = .0000277, *p* < .001), posterior region (β = .0000316, *p* < .001), left hemisphere (β = 3.222 × 10^−5^, *p* < .001), and right hemisphere (β = 2.830 × 10^−5^, *p* = .003).

**Stimulus Effects.** Robust effects of stimulus type were observed for spectral entropy, with consistently higher entropy during Sign Language relative to reversed input. This indicates increased multiscale (broadband) complexity of neural response to linguistically meaningful stimuli. Significant stimulus effects on entropy were found in the frontal cortex (β = –.016, *p* < .001), the posterior cortex (β = –.014, *p* < .001), left hemisphere (β = –.019, *p* = .016), and right hemisphere (β = –.016, *p* = .037). Additionally, spectral spread and centroid in the left hemisphere were modulated by stimulus type (β = –.436, *p* = .032; β = –.683, *p* = .027), reinforcing the left hemisphere’s sensitivity to linguistic features.

The regional specificity of age-related patterns was further confirmed by significant Age × ROI interactions in omnibus models for Spectral Entropy, Centroid, and Spread (all *p* < .001; S2 Table), indicating that age-related trajectories differ substantially across scalp regions and validating the region-specific analytical approach [40].

The omnibus model for Spectral Flatness additionally revealed a significant Age × Stimulus interaction ($\chi^{2}$ = 4.091, *p* = .043), such that the age-related increase in spectral flatness was attenuated during Sign Language relative to Reversed video viewing ($\hat{\beta }$ = −5.856 × 10^−6^). This distributed effect, detectable only at the whole-scalp level, suggests that structured linguistic input partially counteracts age-related spectral dedifferentiation, consistent with experience-dependent stabilization of neural signal organization.

### 4.4. Feature coherence analysis results

**Fig 3:**
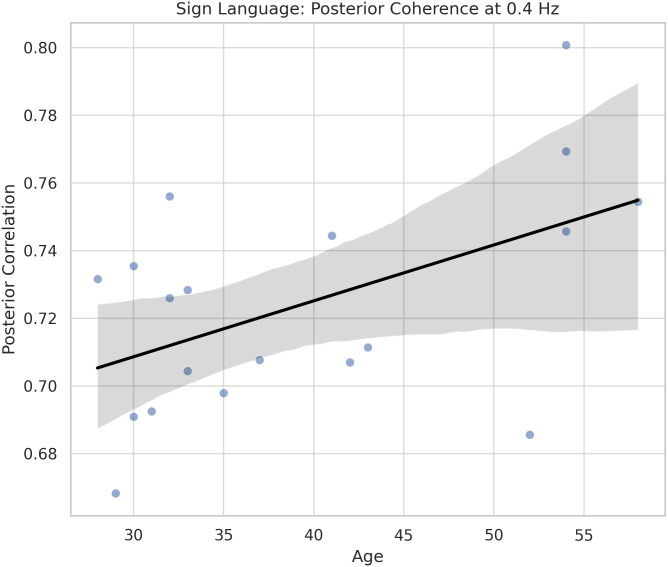
Frontal neural response latency increases with age during non-linguistic visual motion processing. Scatter plot showing a significant positive correlation between participant age and frontal timeshift at 0.2 Hz in the time-reversed video condition (linear fit with 95% confidence interval shown).

Correlation analyses revealed distinct age-related effects across experimental conditions. In the reversed video condition (Condition 0), frontal timeshift at 0.2 Hz exhibited a significant positive correlation with participant age (*r* = .52, *p* = .023; Fig 3). This finding indicates that older participants showed delayed neural response timing to non-linguistic visual motion input.

**Fig 4:**
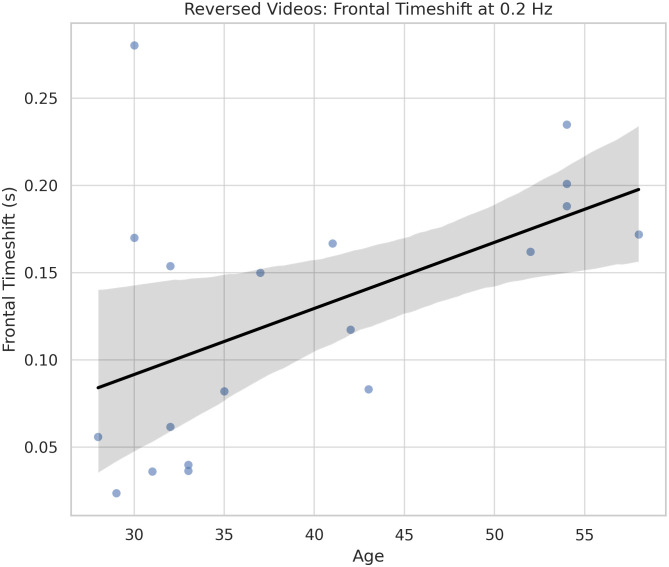
Posterior neural coherence to sign language increases with age. Scatter plot showing a significant positive correlation between participant age and posterior EEG coherence at 0.4 Hz in the sign language condition (linear fit with 95% confidence interval shown).

Conversely, in the Sign Language condition (Condition 1), coherence in the posterior cortex at 0.4 Hz was significantly positively correlated with age (*r* = .51, *p* = .025; Fig 4). This suggests that older participants demonstrated increased neural synchronization with structured linguistic visual motion, consistent with experience-driven predictive processing.

Figs 3 and 4 illustrate representative features at low frequencies, which, based on our prior work ([41]), reflect the temporal scale of predictive processing in visual language comprehension. In the reversed video condition, older participants exhibited longer frontal timeshifts at 0.2 Hz—indicating delayed entrainment to unpredictable, non-linguistic visual input. In contrast, in the sign language condition, coherence in posterior regions (notably including parietal cortex—implicated in motion tracking and visual feature selection; see [42]) increased with age. This pattern suggests that older signers exhibit enhanced entrainment to predictable visual structure in linguistic stimuli, consistent with experience-driven refinement of predictive coding mechanisms.

## 5. Discussion

The present study examined how age and experience influence neural coherence with visual motion during sign language comprehension. We used spectral EEG features and machine learning to assess the impact of age on frequency-specific coherence between brain activity and dynamic visual features, characteristic of linguistic complexity in sign language stimuli [31,37]. Our findings reveal that age and stimulus type exert independent effects on the spectral properties of neural signals. Notably, we observed no significant Age × Stimulus interactions in any spectral measure across scalp regions. This dissociation suggests that the influence of age on EEG spectral features—such as increased spectral flatness—is likely structural in nature, reflecting general neurophysiological changes associated with aging (e.g., reduced neural differentiation and increased noise; [25]). In contrast, the effects of stimulus type, particularly increased spectral entropy during sign language input, appear to be functional, reflecting task- and content-specific engagement of predictive mechanisms in response to structured linguistic input. These findings extend a preliminary analysis of the present dataset [39] by adding spectral analysis across brain regions and linear mixed-effects modeling to characterize ageing trajectories of predictive neural mechanisms.

The distinction between processing of linguistic vs. non-linguistic input is further supported by our coherence analyses, which examined the entrainment of neural activity to visual motion (optical flow) in the stimuli. While spectral features provided a global view of neural signal organization, coherence measures offered a more dynamic, stimulus-locked window into predictive processing. Here, age effects were modulated by brain region and condition: in non-linguistic reversed videos, older participants exhibited delayed entrainment as indexed by frontal electrodes, suggesting difficulty integrating unpredictable sensory input with internal models. Conversely, in the sign language condition, age was positively associated with posterior coherence strength, particularly at low frequencies associated with phrasal or supra-lexical timescales. This finding points to a refinement of predictive mechanisms with lifelong language experience—consistent with hierarchical inference models under the Free Energy Principle [1].

Crucially, activity recorded over posterior scalp regions (likely with contributions from parietal cortex linked to motion tracking and visual attention) highlights the visual pathway’s sensitivity to linguistic structure in signers. Recent work [42] has shown that these regions play a central role in encoding rhythmic structure in biological motion, further supporting their role in sign language comprehension. Our results suggest that with increased experience, the visual system becomes finely tuned to the temporal regularities of linguistic motion, allowing for more efficient and anticipatory neural responses. This trajectory is consistent with behavioral evidence that early-stage sign language learners (1.5–2 years of experience) initially show enhanced visual temporal resolution, which later normalizes as predictive processing mechanisms take over at higher proficiency level [43].

Together, these findings suggest a dual model of aging and language processing: while structural neural changes (e.g., increased spectral flatness) reflect general aging effects, functional entrainment to linguistic input remains robust (and even enhanced) in experienced signers. While the present data do not directly demonstrate that older signers generate more accurate predictions or show reduced prediction error during comprehension, our findings are consistent with the hypothesis that lifelong linguistic experience refines the neural mechanisms that support prediction, as evidenced by enhanced posterior coherence specifically during processing of structured linguistic motion.

It is interesting that the whole-scalp omnibus model revealed a significant Age × Stimulus interaction for Spectral Flatness, such that the age-related increase in spectral flatness was attenuated during Sign Language relative to Reversed video viewing. This distributed effect, detectable only by pooling across scalp regions, suggests that structured linguistic input might partially counteract age-related spectral dedifferentiation at the whole-brain level. This is consistent with the experience-dependent compensation account: while aging increases spectral uniformity globally, engagement with familiar linguistic structure modulates this trajectory, pointing to functional stabilization of neural signal organization during predictive linguistic processing.

This implies that language experience supports compensatory predictive mechanisms, sustaining efficient comprehension despite broader age-related declines in structural organization. The use of both spectral and coherence-based analyses thus provides a comprehensive view of how individual trajectories of aging and experience jointly shape neural processing in the visual linguistic domain.

Our findings support the hypothesis that life-long exposure to structured visual language modulates the temporal and spatial dynamics of neural prediction [44,45].

In the reversed video condition, older participants showed longer frontal timeshifts at low frequencies (0.2 Hz), indicating delayed response to non-linguistic visual motion. This pattern is consistent with age-related dedifferentiation in frontal cortical function, where predictive engagement is limited in the absence of structured input [45,46]. In contrast, in the Sign Language condition, coherence in posterior regions at 0.4 Hz positively correlated with age. This suggests that older signers engage in enhanced low-frequency predictive processing during linguistic input, consistent with hierarchical inference over longer temporal windows [24,41].

These results align with the Free Energy Principle (FEP) framework [1], which posits that the brain reduces sensory surprise by forming internal generative models. In the context of sign language, increased posterior coherence with age may reflect improved capacity to anticipate upcoming linguistic events based on prior experience [23]. The frequency range of this coherence (0.4 Hz) corresponds to supra-lexical linguistic units, suggesting that predictive mechanisms operate at the phrasal or clause level [5].

The specificity of this age-related increase (which occurs only for linguistically structured motion and is localized to posterior regions associated with visual motion analysis) reflects experience-dependent optimization of visual predictive models rather than generic age-related compensatory processing. If the enhanced coherence simply reflected general compensatory mechanisms, we would expect similar increases for time-reversed stimuli or frontal regions. Instead, the selective enhancement during sign language processing in posterior cortex points to refinement of domain-specific internal models that have been shaped by decades of exposure to the statistical structure of visual language.

Comparisons with prior studies in the auditory domain show parallel age-related trends in predictive processing. Increased spectral flatness across regions in older participants replicates known effects of reduced neural complexity with age, and mirrors findings in speech comprehension where older adults rely more heavily on top-down predictive mechanisms [25].

All participants in this study were proficient and fluent signers of ÖGS. Language competence, like healthy aging, is an inherently individual-level trait rather than a categorical group-level variable. Accordingly, each participant’s data represent a valid realization of how linguistic processing and neural coherence patterns are shaped by their unique experience profile, rather than sampling error around a group mean. Our analytical framework explicitly accounts for this by employing linear mixed-effects models (LMMs) with participant-level random effects, allowing us to model both within- and between-participant variability in neural coherence measures. This approach aligns with established psycholinguistic research practices that treat individual-level differences as theoretically meaningful rather than statistical noise [47]. While the cross-sectional design in the present study limits inferences about individual aging trajectories, the present analysis nonetheless reveals systematic, experience-dependent patterns of neural coherence that are robust to inter-individual variability. Longitudinal data would be necessary to directly capture how predictive neural dynamics evolve over time within individuals; however, the current results offer an important cross-sectional snapshot of age-related shifts in predictive processing within an experienced signer population. While our cross-sectional age comparisons combined with condition contrasts provide evidence consistent with experience-dependent refinement of predictive mechanisms, future work using direct manipulations of predictability within sign language stimuli (e.g., varying lexical frequency, typical age of acquisition for signs, or syntactic predictability) could provide more direct evidence for how these mechanisms operate in experienced signers.

Future studies should use longitudinal designs and include measures of working memory, attention, and language proficiency to disentangle the contributions of general cognitive versus language-specific mechanisms. Extension to hearing L2 sign language learners and bimodal bilinguals could further illuminate how experience shapes the predictive brain across modalities and populations.

## 6. Conclusion

The data and results of analysis in the present study support the hypothesis that life-long visual language experience refines predictive coding in the brain. The transition from sensory-driven to model-driven inference appears to be reflected in frequency-specific coherence dynamics, particularly in frontal and posterior regions. These findings contribute to a growing body of evidence that predictive processing is shaped by experience, and that sign language provides a powerful lens into these mechanisms in the visual modality.

## Supporting information

S1 Table Region-specific linear mixed model results (Tables S1a–S1p). Complete fixed effects estimates and Type II likelihood ratio tests for all 16 region-specific models (4 spectral features × 4 ROIs).
(PDF)

S2 Table Omnibus linear mixed model results (Tables S2a–S2d). Fixed effects estimates and Type II likelihood ratio tests for omnibus models including ROI as a fixed effect predictor, fitted separately for each of the four spectral features.
(PDF)

S1 File Inclusivity in global research form. Authors’ responses to the PLOS ONE Inclusivity in Global Research questionnaire.
(DOCX)
