# Supplementary material for: Age and language experience modulate predictive processing in the visual modality
Source: PLoS One. 2026 May 15;21(5):e0346695. doi: 10.1371/journal.pone.0346695 (PMC13178858; doi:10.1371/journal.pone.0346695)
Supplement: S1 Table — Complete fixed effects estimates and Type II likelihood ratio tests for all 16 region-specific models (4 spectral features × 4 ROIs). (PDF) [file pone.0346695.s001.pdf]

Supporting information

Table S1. All 16 models (4 spectral features × 4 ROIs) were fitted using maximum likelihood (ML) estimation with Type II likelihood ratio tests for fixed effects. Each model was specified as:

$$f_{ij} = \beta_0 + \beta_1(\text{Age}_i) + \beta_2(\text{Stimulus}_j) + \beta_3(\text{Age}_i \times \text{Stimulus}_j) + u_i + \varepsilon_{ij}$$

where f_{ij} is the spectral feature value for participant i in stimulus condition j; β_0 is the fixed intercept representing the unweighted grand mean across all participants;β_1 is the fixed effect of Age; β_2 is the fixed effect of Stimulus; β_3 is the Age × Stimulus interaction; u_i is the participant-specific random intercept, capturing individual deviations from the grand mean; and ε_{ij} is the residual error. No random slopes were modeled. Stimulus was treatment-coded with Reversed video as the reference level (coded 0), such that β_2 reflects the deviation of Sign Language relative to Reversed. The intercept corresponds to the unweighted grand mean under sum contrast coding.

Table S1a. Linear Mixed Model Results: Spectral Centroid, Right Hemisphere

Panel A: Likelihood Ratio Tests (Type II)

Effect	*df*	χ^2	*p*
Age	1	0.003	.957
Stimulus	1	16.946	<.001
Age × Stimulus	1	0.490	.484

Panel B: Fixed Effects Estimates

Term	$\hat{\beta}$	SE	*t*	*p*
Intercept	7.845	3.200	2.451	.024
Age	0.004	0.079	0.054	.958
Stimulus [SL]	−0.466	0.276	−1.687	.092
Age × Stimulus [SL]	0.005	0.007	0.700	.484

Table S1b. Linear Mixed Model Results: Spectral Centroid, Frontal Region

Panel A: Likelihood Ratio Tests (Type II)

Effect	*df*	χ^2	*p*
Age	1	0.040	.842
Stimulus	1	15.482	<.001
Age × Stimulus	1	0.703	.402

Panel B: Fixed Effects Estimates

Term	$\hat{\beta}$	SE	*t*	*p*
Intercept	7.205	3.061	2.354	.029
Age	0.015	0.075	0.200	.844
Stimulus [SL]	−0.526	0.296	−1.777	.076
Age × Stimulus [SL]	0.006	0.007	0.839	.402

Table S1c. Linear Mixed Model Results: Spectral Centroid, Left Hemisphere

Panel A: Likelihood Ratio Tests (Type II)

Effect	*df*	χ^2	*p*
Age	1	0.981	.322
Stimulus	1	25.464	<.001
Age × Stimulus	1	1.024	.312

Panel B: Fixed Effects Estimates

Term	$\hat{\beta}$	SE	*t*	*p*
Intercept	5.252	2.917	1.800	.088
Age	0.072	0.072	1.003	.328
Stimulus [SL]	−0.683	0.308	−2.220	.027
Age × Stimulus [SL]	0.008	0.008	1.012	.312

Table S1d. Linear Mixed Model Results: Spectral Centroid, Posterior Region

Panel A: Likelihood Ratio Tests (Type II)

Effect	*df*	χ^2	*p*
Age	1	0.904	.342
Stimulus	1	21.433	<.001
Age × Stimulus	1	0.116	.733

Panel B: Fixed Effects Estimates

Term	$\hat{\beta}$	SE	*t*	*p*
Intercept	5.463	2.730	2.001	.060
Age	0.065	0.067	0.962	.348
Stimulus [SL]	−0.413	0.282	−1.465	.143
Age × Stimulus [SL]	0.002	0.007	0.341	.734

Table S1e. Linear Mixed Model Results: Spectral Entropy, Right Hemisphere

Panel A: Likelihood Ratio Tests (Type II)

Effect	*df*	χ^2	*p*
Age	1	0.066	.798
Stimulus	1	21.191	<.001
Age × Stimulus	1	0.987	.320

Panel B: Fixed Effects Estimates

Term	$\hat{\beta}$	SE	*t*	*p*
Intercept	0.533	0.075	7.084	<.001
Age	-4.744×10^{-4}	0.002	−0.256	.801
Stimulus [SL]	−0.016	0.008	−2.092	.037
Age × Stimulus [SL]	1.865×10^{-4}	1.876×10^{-4}	0.994	.320

Table S1f. Linear Mixed Model Results: Spectral Entropy, Frontal Region

Panel A: Likelihood Ratio Tests (Type II)

Effect	*df*	χ^2	*p*
Age	1	0.233	.629
Stimulus	1	17.894	<.001
Age × Stimulus	1	1.121	.290

Panel B: Fixed Effects Estimates

Term	$\hat{\beta}$	SE	*t*	*p*
Intercept	0.479	0.065	7.350	<.001
Age	7.769×10^{-4}	0.002	0.484	.634
Stimulus [SL]	−0.016	0.008	−2.063	.039
Age × Stimulus [SL]	2.047×10^{-4}	1.934×10^{-4}	1.059	.290

Table S1g. Linear Mixed Model Results: Spectral Entropy, Left Hemisphere

Panel A: Likelihood Ratio Tests (Type II)

Effect	*df*	χ^2	*p*
Age	1	1.278	.258
Stimulus	1	38.682	<.001
Age × Stimulus	1	0.843	.358

Panel B: Fixed Effects Estimates

Term	$\hat{\beta}$	SE	*t*	*p*
Intercept	0.454	0.059	7.729	<.001
Age	0.002	0.001	1.150	.264
Stimulus [SL]	−0.019	0.008	−2.420	.016
Age × Stimulus [SL]	1.751×10^{-4}	1.906×10^{-4}	0.918	.359

Table S1h. Linear Mixed Model Results: Spectral Entropy, Posterior Region

Panel A: Likelihood Ratio Tests (Type II)

Effect	*df*	χ^2	*p*
Age	1	0.907	.341
Stimulus	1	22.743	<.001
Age × Stimulus	1	0.601	.438

Panel B: Fixed Effects Estimates

Term	$\hat{\beta}$	SE	*t*	*p*
Intercept	0.469	0.058	8.016	<.001
Age	0.001	0.001	0.964	.347
Stimulus [SL]	−0.014	0.007	−1.921	.055
Age × Stimulus [SL]	1.401×10^{-4}	1.806×10^{-4}	0.775	.438

Table S1i. Linear Mixed Model Results: Spectral Flatness, Right Hemisphere

Panel A: Likelihood Ratio Tests (Type II)

Effect	*df*	χ^2	*p*
Age	1	8.639	.003
Stimulus	1	1.050	.306
Age × Stimulus	1	1.035	.309

Panel B: Fixed Effects Estimates

Term	$\hat{\beta}$	SE	*t*	*p*
Intercept	0.001	3.474×10^{-4}	3.165	.005
Age	2.830×10^{-5}	8.556×10^{-6}	3.307	.004
Stimulus [SL]	1.786×10^{-4}	2.426×10^{-4}	0.736	.462
Age × Stimulus [SL]	-6.080×10^{-6}	5.975×10^{-6}	−1.018	.309

Table S1j. Linear Mixed Model Results: Spectral Flatness, Frontal Region

Panel A: Likelihood Ratio Tests (Type II)

Effect	*df*	χ^2	*p*
Age	1	11.299	<.001
Stimulus	1	2.724	.099
Age × Stimulus	1	1.142	.285

Panel B: Fixed Effects Estimates

Term	$\hat{\beta}$	SE	*t*	*p*
Intercept	0.001	2.766×10^{-4}	3.740	.001
Age	2.677×10^{-5}	6.813×10^{-6}	3.929	<.001
Stimulus [SL]	1.437×10^{-4}	2.270×10^{-4}	0.633	.527
Age × Stimulus [SL]	-5.978×10^{-6}	5.592×10^{-6}	−1.069	.285

Table S1k. Linear Mixed Model Results: Spectral Flatness, Left Hemisphere

Panel A: Likelihood Ratio Tests (Type II)

Effect	*df*	χ^2	*p*
Age	1	11.057	<.001
Stimulus	1	0.675	.411
Age × Stimulus	1	0.670	.413

Panel B: Fixed Effects Estimates

Term	$\hat{\beta}$	SE	*t*	*p*
Intercept	8.589×10^{-4}	3.378×10^{-4}	2.543	.020
Age	3.222×10^{-5}	8.320×10^{-6}	3.873	.001
Stimulus [SL]	1.395×10^{-4}	2.352×10^{-4}	0.593	.553
Age × Stimulus [SL]	-4.743×10^{-6}	5.793×10^{-6}	−0.819	.413

Table S1l. Linear Mixed Model Results: Spectral Flatness, Posterior Region

Panel A: Likelihood Ratio Tests (Type II)

Effect	*df*	χ^2	*p*
Age	1	10.888	<.001
Stimulus	1	1.863	.172
Age × Stimulus	1	1.288	.256

Panel B: Fixed Effects Estimates

Term	$\hat{\beta}$	SE	*t*	*p*
Intercept	9.377×10^{-4}	3.348×10^{-4}	2.801	.011
Age	3.162×10^{-5}	8.246×10^{-6}	3.834	.001
Stimulus [SL]	1.817×10^{-4}	2.370×10^{-4}	0.767	.443
Age × Stimulus [SL]	-6.624×10^{-6}	5.836×10^{-6}	−1.135	.257

Table S1m. Linear Mixed Model Results: Spectral Spread, Right Hemisphere

Panel A: Likelihood Ratio Tests (Type II)

Effect	*df*	χ^2	*p*
Age	1	0.268	.605
Stimulus	1	2.030	.154
Age × Stimulus	1	0.657	.418

Panel B: Fixed Effects Estimates

Term	$\hat{\beta}$	SE	*t*	*p*
Intercept	9.679	2.055	4.711	<.001
Age	0.026	0.051	0.519	.610
Stimulus [SL]	−0.216	0.191	−1.134	.257
Age × Stimulus [SL]	0.004	0.005	0.811	.418

Table S1n. Linear Mixed Model Results: Spectral Spread, Frontal Region

Panel A: Likelihood Ratio Tests (Type II)

Effect	*df*	χ^2	*p*
Age	1	0.155	.693
Stimulus	1	2.929	.087
Age × Stimulus	1	1.667	.197

Panel B: Fixed Effects Estimates

Term	$\hat{\beta}$	SE	*t*	*p*
Intercept	10.048	2.194	4.580	<.001
Age	0.021	0.054	0.395	.697
Stimulus [SL]	−0.343	0.205	−1.671	.095
Age × Stimulus [SL]	0.007	0.005	1.291	.197

Table S1o. Linear Mixed Model Results: Spectral Spread, Left Hemisphere

Panel A: Likelihood Ratio Tests (Type II)

Effect	*df*	χ^2	*p*
Age	1	1.501	.221
Stimulus	1	4.746	.029
Age × Stimulus	1	2.767	.096

Panel B: Fixed Effects Estimates

Term	$\hat{\beta}$	SE	*t*	*p*
Intercept	8.232	2.074	3.969	<.001
Age	0.064	0.051	1.250	.227
Stimulus [SL]	−0.436	0.203	−2.147	.032
Age × Stimulus [SL]	0.008	0.005	1.664	.096

Table S1p. Linear Mixed Model Results: Spectral Spread, Posterior Region

Panel A: Likelihood Ratio Tests (Type II)

Effect	*df*	χ^2	*p*
Age	1	1.159	.282
Stimulus	1	4.892	.027
Age × Stimulus	1	0.805	.370

Panel B: Fixed Effects Estimates

Term	$\hat{\beta}$	SE	*t*	*p*
Intercept	8.388	1.992	4.210	<.001
Age	0.054	0.049	1.093	.288
Stimulus [SL]	−0.254	0.180	−1.411	.158
Age × Stimulus [SL]	0.004	0.004	0.897	.370
