# Supplementary material for: Age and language experience modulate predictive processing in the visual modality
Source: PLoS One. 2026 May 15;21(5):e0346695. doi: 10.1371/journal.pone.0346695 (PMC13178858; doi:10.1371/journal.pone.0346695)
Supplement: S2 Table — Fixed effects estimates and Type II likelihood ratio tests for omnibus models including ROI as a fixed effect predictor, fitted separately for each of the four spectral features. (PDF) [file pone.0346695.s002.pdf]

Table S2.

The 4 models (one for each spectral parameter) are fitted using maximum likelihood (ML). Type II likelihood ratio tests used for omnibus effects. The omnibus model was specified as $f_{ijk} = \beta_0 + \beta_1(\text{Age}_i) + \beta_2(\text{Stimulus}_j) + \beta_3(\text{ROI}_k) + \beta_4(\text{Age}_i \times \text{Stimulus}_j) + \beta_5(\text{Age}_i \times \text{ROI}_k) + \beta_6(\text{Stimulus}_j \times \text{ROI}_k) + \beta_7(\text{Age}_i \times \text{Stimulus}_j \times \text{ROI}_k) + u_i + \varepsilon_{ijk}$, where f_{ijk} is the spectral feature for participant i, stimulus condition j, and region k; u_i is the random intercept for participant i; and ε_{ijk} is the residual error. Random intercepts included for subject. Stimulus was treatment-coded with Reversed as the reference level; the Stimulus coefficient reflects the deviation of Sign Language from Reversed. ROI was sum-coded; ROI coefficients reflect deviations of Posterior, Left, and Right from the grand mean across all four regions, with Frontal as the implicit omitted category. Random intercepts included for subject.

Table S2a. Omnibus Linear Mixed Model Results for Spectral Centroid

Panel A: Likelihood Ratio Tests (Type II)

Effect	*df*	χ^2	*p*
Age	1	0.304	.581
Stimulus	1	71.244	<.001
ROI	3	8.380	.039
Age × Stimulus	1	1.946	.163
Age × ROI	3	62.604	<.001
Stimulus × ROI	3	1.198	.754
Age × Stimulus × ROI	3	0.270	.966

Panel B: Fixed Effects Estimates

Term	$\hat{\beta}$	SE	*t*	*p*
Intercept	6.441	2.862	2.251	.036
Age	0.039	0.070	0.554	.586
Stimulus [SL]	−0.522	0.152	−3.432	<.001
ROI [Posterior]	0.764	0.264	2.899	.004
ROI [Left]	−1.190	0.264	−4.515	<.001
ROI [Right]	−0.978	0.264	−3.710	<.001
Age × Stimulus [SL]	0.005	0.004	1.395	.163
Age × ROI [Posterior]	−0.024	0.006	−3.691	<.001
Age × ROI [Left]	0.033	0.006	5.092	<.001
Age × ROI [Right]	0.026	0.006	3.957	<.001
Stimulus [SL] × ROI [Posterior]	−0.004	0.264	−0.016	.988
Stimulus [SL] × ROI [Left]	−0.161	0.264	−0.611	.541
Stimulus [SL] × ROI [Right]	0.109	0.264	0.412	.680
Age × Stimulus [SL] × ROI [Posterior]	<0.001	0.006	0.137	.891
Age × Stimulus [SL] × ROI [Left]	0.002	0.006	0.377	.706
Age × Stimulus [SL] × ROI [Right]	−0.003	0.006	−0.441	.659

Table S2b. Omnibus Linear Mixed Model Results for Spectral Entropy

Panel A: Likelihood Ratio Tests (Type II)

Effect	*df*	χ^2	*p*
Age	1	0.326	.568
Stimulus	1	85.910	<.001
ROI	3	30.014	<.001
Age × Stimulus	1	3.112	.078
Age × ROI	3	67.298	<.001
Stimulus × ROI	3	2.232	.526
Age × Stimulus × ROI	3	0.056	.997

Panel B: Fixed Effects Estimates

Term	$\hat{\beta}$	SE	*t*	*p*
Intercept	0.484	0.059	8.136	<.001
Age	8.388×10^{-4}	1.464×10^{-3}	0.573	.573
Stimulus [SL]	−0.016	0.004	−3.997	<.001
ROI [Posterior]	−0.005	0.007	−0.701	.483
ROI [Left]	−0.029	0.007	−4.188	<.001
ROI [Right]	−0.015	0.007	−2.078	.038
Age × Stimulus [SL]	1.766×10^{-4}	1.001×10^{-4}	1.764	.078
Age × ROI [Posterior]	-6.192×10^{-5}	1.734×10^{-4}	−0.357	.721
Age × ROI [Left]	8.248×10^{-4}	1.734×10^{-4}	4.758	<.001
Age × ROI [Right]	5.503×10^{-4}	1.734×10^{-4}	3.174	.002
Stimulus [SL] × ×ROI [Posterior]	4.111×10^{-5}	0.007	0.006	.995
Stimulus [SL] × × ROI [Left]	−0.002	0.007	−0.354	.724
Stimulus [SL] × × ROI [Right]	0.002	0.007	0.305	.760
Age × Stimulus [SL] × × ROI [Posterior]	2.815×10^{-5}	1.734×10^{-4}	0.162	.871
Age × Stimulus [SL] × × ROI [Left]	-1.518×10^{-6}	1.734×10^{-4}	−0.009	.993
Age × Stimulus [SL] × × ROI [Right]	-3.653×10^{-5}	1.734×10^{-4}	−0.211	.833

Table S2c. Omnibus Linear Mixed Model Results for Spectral Flatness

Panel A: Likelihood Ratio Tests (Type II)

Effect	*df*	χ^2	*p*
Age	1	12.660	<.001
Stimulus	1	5.879	.015
ROI	3	2.835	.418
Age × Stimulus	1	4.091	.043
Age × ROI	3	0.615	.893
Stimulus × ROI	3	0.350	.950
Age × Stimulus × ROI	3	0.057	.996

Panel B: Fixed Effects Estimates

Term	$\hat{\beta}$	SE	*t*	*p*
Intercept	9.826×10^{-4}	2.845×10^{-4}	3.454	.003
Age	2.973×10^{-5}	7.008×10^{-6}	4.242	<.001
Stimulus [SL]	1.609×10^{-4}	1.175×10^{-4}	1.369	.171
ROI [Posterior]	5.179×10^{-5}	2.036×10^{-4}	0.254	.799
ROI [Left]	-1.236×10^{-4}	2.036×10^{-4}	−0.607	.544
ROI [Right]	-4.489×10^{-5}	2.036×10^{-4}	−0.220	.825
Age × Stimulus [SL]	-5.856×10^{-6}	2.895×10^{-6}	−2.023	.043
Age × ROI [Posterior]	-2.960×10^{-6}	5.014×10^{-6}	−0.590	.555
Age × ROI [Left]	2.498×10^{-6}	5.014×10^{-6}	0.498	.618
Age × ROI [Right]	1.891×10^{-6}	5.014×10^{-6}	0.377	.706
Stimulus [SL] × × ROI [Posterior]	-1.717×10^{-5}	2.036×10^{-4}	−0.084	.933
Stimulus [SL] × ×ROI [Left]	-2.139×10^{-5}	2.036×10^{-4}	−0.105	.916
Stimulus [SL] × ×ROI [Right]	2.083×10^{-5}	2.036×10^{-4}	0.102	.918
Age × Stimulus [SL] × × ROI [Posterior]	-1.215×10^{-7}	5.014×10^{-6}	−0.024	.981
Age × Stimulus [SL] × × ROI [Left]	1.113×10^{-6}	5.014×10^{-6}	0.222	.824
Age × Stimulus [SL] × × ROI [Right]	-7.679×10^{-7}	5.014×10^{-6}	−0.153	.878

Table S2d. Omnibus Linear Mixed Model Results for Spectral Spread

Panel A: Likelihood Ratio Tests (Type II)

Effect	*df*	χ^2	*p*
Age	1	0.694	.405
Stimulus	1	12.620	<.001
ROI	3	30.405	<.001
Age × Stimulus	1	5.016	.025
Age × ROI	3	50.089	<.001
Stimulus × ROI	3	0.383	.944
Age × Stimulus × ROI	3	0.555	.907

Panel B: Fixed Effects Estimates

Term	$\hat{\beta}$	SE	*t*	*p*
Intercept	9.087	1.994	4.558	<.001
Age	0.041	0.049	0.841	.411
Stimulus [SL]	−0.312	0.103	−3.045	.002
ROI [Posterior]	0.961	0.178	5.412	<.001
ROI [Left]	−0.855	0.178	−4.814	<.001
ROI [Right]	−0.698	0.178	−3.932	<.001
Age × Stimulus [SL]	0.006	0.003	2.240	.025
Age × ROI [Posterior]	−0.020	0.004	−4.554	<.001
Age × ROI [Left]	0.023	0.004	5.157	<.001
Age × ROI [Right]	0.012	0.004	2.826	.005
Stimulus [SL] × ROI [Posterior]	−0.031	0.178	−0.174	.862
Stimulus [SL] × ROI [Left]	−0.124	0.178	−0.698	.485
Stimulus [SL] × ROI [Right]	0.059	0.178	0.330	.741
Age × Stimulus [SL] × ROI [Posterior]	8.727×10^{-4}	0.004	0.199	.842
Age × Stimulus [SL] × ROI [Left]	0.003	0.004	0.610	.542
Age × Stimulus [SL] × ROI [Right]	−0.002	0.004	−0.386	.700
