# Supplementary material for: Age and language experience modulate predictive processing in the visual modality
Source: PLoS One. 2026 May 15;21(5):e0346695. doi: 10.1371/journal.pone.0346695 (PMC13178858; doi:10.1371/journal.pone.0346695)
Supplement: S1 File — Authors’ responses to the PLOS ONE Inclusivity in Global Research questionnaire. (DOCX) [file pone.0346695.s003.docx]

Inclusivity in global research

PLOS’ policy on inclusivity in global research aims to improve transparency in the reporting of research performed outside of researchers’ own country or community and ensures that PLOS publications reporting global research adhere to high standards for research ethics and authorship. Authors of relevant research articles may be asked to complete the questionnaire below, which outlines ethical, cultural, and scientific considerations specific to inclusivity in global research. This questionnaire may be requested when researchers have travelled to a different country to conduct research, if research uses samples collected in another country, research with Indigenous populations or their lands, or if research is on cultural artefacts. Researchers travelling to another country solely to use laboratory equipment will not normally be required to complete the questionnaire. However, the questionnaire can be requested at the journal’s discretion for any submission – if you have been requested to complete this questionnaire by the PLOS journal you submitted to, please do so.

Please complete the questionnaire below and include this as a Supporting Information file with your manuscript. Note that if your paper is accepted for publication, this checklist will be published with your article in the supporting information files. Please ensure that you reference the checklist in the main body of your manuscript. We suggest adding a subsection ‘Inclusivity in global research’ to your Methods section and adding the following sentence: “Additional information regarding the ethical, cultural, and scientific considerations specific to inclusivity in global research is included in the Supporting Information (SX Checklist)”

The questions have been designed to be applicable to a wide range of study types, and there are subsections for both human subjects research and non-human subjects research. If any of the questions are not relevant to your research please mark them as “N/A” as appropriate.

**Ethical considerations, permits and authorship**

*This section is applicable to all research types.*

Provide details as to who granted permissions and/or consent for the study to take place in the Methods section of your manuscript. This should include the names of **all** ethics boards, governmental organizations, community leaders or other bodies that provided approval for the study. If individuals provided approval refer to these people by their role or title but do not list their name(s).

Reported on page number: 5, lines 174-177

If there were any deviations from the study protocol after approval was obtained please provide details of these changes in the Methods section of your manuscript.
Did this study involve local collaborators that are residents of the country where the research was conducted or members of the community studied? If you do not have any authors from said communities, please provide an explanation for this below.

Reported on page number: N/A

The Austrian Deaf community is an essential collaborator for our research team. Without their support, research on Austrian Sign Language (ÖGS) would not be possible. Deaf signing experts are involved in our studies at the conceptualization stage, providing crucial information about the grammatical structure of ÖGS. This input is indispensable, as many aspects of ÖGS grammar remain underexplored, and understanding the relevant grammatical rules is a prerequisite for investigating the neural processing of specific linguistic structures.

However, participation in the form of providing grammatical information alone does not meet the criteria for authorship as defined in the scientific context (e.g., Albert & Wager, 2003; Osborne & Holland, 2009).

One of the authors, Julia Krebs, is an ÖGS signer. She completed ÖGS interpreter training in 2009 and worked as an interpreter for several years. Julia Krebs maintains regular contact with members of the Austrian Deaf community and collaborates with Deaf researchers on other projects. Unfortunately, there are currently no Deaf researchers in Austria working on the experimental investigation of the neural processing of ÖGS. We hope that this will change in the future.

**References:**

Albert, T., & Wager, E. (2003). How to handle authorship disputes: A guide for new researchers. *The COPE Report*. <http://publicationethics.org/files/u2/2003pdf12.pdf>.

Osborne, J. W., & Holland, A. (2009). What is authorship, and what should it be? A survey of prominent guidelines for determining authorship in scientific publications. *Practical Assessment, Research, and Evaluation*, *14*(1).

Everyone listed as an author should meet PLOS’ criteria for authorship and all individuals who meet these criteria should be included in the author byline, rather than the acknowledgements. For further information please see the journal’s Authorship Policy.

**Human subjects research (e.g. health research, medical research, cross-cultural psychology)**

Did you obtain written informed consent from a representative of the local community or region before the research took place? How did you establish who speaks for the community? Details of written informed consent obtained from study participants should be reported separately in the Methods section of your manuscript.

Our research group is in regular exchange with the Austrian Deaf community. We have established a long-standing relationship with the community, and a group of Deaf signers has been supporting our work for several years.

We have added information about informed consent to the Methods section of the manuscript.

How did members of the local community provide input on the aims of the research investigation, its methodology, and its anticipated outcome(s)?

The Deaf signers acted as linguistic informants, contributing essential insights into the grammatical structure of ÖGS. Accordingly, their involvement took place at the conceptual level of this study.

When engaging with the local community, how did you ensure that the informed consent documents and other materials could be understood by local stakeholders?

During the time the Deaf participants were in our lab, an ÖGS signer (Julia Krebs) was always present.

Informed consent was obtained in written form. The content of the consent form was summarized in ÖGS for all participants and further explained in ÖGS whenever participants had additional questions.

Will the findings of the research be made available in an understandable format to stakeholders in the community where the study was conducted (e.g. via a presentation, summary report, copies of publications, etc.)? Please provide details of how this will be achieved.

As before, we will make our research findings accessible to the Austrian Deaf community in an understandable format. We give presentations not only in scientific settings but also in non-academic contexts—either directly in ÖGS (by Julia Krebs) or with the support of interpreters.

We also publish summary reports in written German, for example in journals related to Deafness and sign languages, such as *Das Zeichen. Zeitschrift für Sprache und Kultur Gehörloser* (<https://www.das-zeichen.online/>).

For us, it is important to give back to the community.
